# Evaluation of adrenal tumors and analysis of the metabolic profile of patients with incidentaloma

**DOI:** 10.1590/0100-6991e-20243685-en

**Published:** 2024-12-12

**Authors:** PEDRO VICTOR GONÇALVES MONTALVÃO, IURI MOURA MANGUEIRA, GABRIEL DA MOTTA ALVES, JOÃO VITOR FAZZIO CORDEIRO, MARCIA HELENA SOARES COSTA, GUILHERME DE ANDRADE GAGHEGGI RAVANINI

**Affiliations:** 1 - Universidade Federal do Estado do Rio de Janeiro, Medicina - Rio de Janeiro - RJ - Brasil; 2 - Universidade Estadual do Rio de Janeiro, Serviço de Clínica Médica - Rio de Janeiro - RJ - Brasil; 3 - Universidade Federal do Estado do Rio de Janeiro, Departamento de Clínica Médica - Serviço de Endocrinologia HUGG/EBSERH - Rio de Janeiro - RJ - Brasil; 4 - Universidade Federal do Estado do Rio de Janeiro, Departamento de Cirurgia Geral - Serviço de Cirurgia Oncológica HUGG/EBSERH - Rio de Janeiro - RJ - Brasil

**Keywords:** Adrenocortical Hyperfunction, Adrenocortical Adenoma, Adrenal Gland Neoplasms, Incidental Findings, Adrenal Gland, Hiperfunção Adrenocortical, Adenoma Adrenocortical, Doenças das Glândulas Suprarrenais, Achados Incidentais, Glândulas Suprarrenais

## Abstract

**Introduction:**

Advances in imaging methods have led to an increasingly frequent diagnosis of adrenal gland lesions as incidental findings. Despite progress in this field, there is still limited information regarding the epidemiology of the clinical and metabolic profile of patients with adrenal incidentaloma (AI). The objective is analyze the epidemiology of adrenal tumors at Gaffrée e Guinle University Hospital (HUGG) and compare it with data from the literature.

**Method:**

This is a cross-sectional study that included patients of any gender and age who was treated at HUGG for adrenal tumors.

**Results:**

The following variables were evaluated: age, gender, functionality, benignity, and size. We also analyzed the metabolic profile of patients with AI, specifically those with mild autonomy cortisol secretion. Out of 31 patients with adrenal tumors, 68% were female. The mean age was 55 years with a standard deviation of ±16.2. 54% of the sample had adrenal incidentalomas. 93.6% of the sample had benign cases. Among the adrenal incidentalomas, 53% were non-functioning. In patients with adrenal tumors, only 10% had metabolic syndrome, while in patients with mild autonomous cortisol secretion, this number rose to 17%.

**Conclusion:**

The sample of patients with adrenal tumors and incidentaloma at HUGG presented a prevalence of metabolic complications similar to that found in the literature.

## RESUMO

**Introdução:**

O avanço nos métodos de imagem tem levado a um diagnóstico cada vez mais frequente de lesões das glândulas adrenais como achado incidental. Apesar dos progressos nessa área, ainda há pouca informação sobre a epidemiologia do perfil clínico e metabólico de pacientes com incidentaloma das glândulas adrenais (IG). O objetivo deste estudo é analisar a epidemiologia dos tumores adrenais do Hospital Universitário Gaffrée e Guinle (HUGG) e comparar com os dados da literatura.

**Método:**

Trata-se de um estudo transversal em que foram incluídos pacientes de qualquer sexo e idade atendidos no HUGG com tumores em adrenais.

**Resultados:**

Foram avaliadas as seguintes variáveis: idade, sexo, funcionalidade, benignidade e tamanho. Analisamos também o perfil metabólico de pacientes com IA e mais especificamente daqueles com autonomia de secreção do cortisol. De 31 pacientes com tumores de adrenal, 68% eram do sexo feminino. A idade média foi de 55 anos com desvio padrão de 16,2. 54% da amostra eram incidentalomas de adrenais. 93.6% da amostra eram casos benignos. Dos incidentalomas de adrenal, 53% eram não secretores. Nos pacientes com tumores de adrenal apenas 10% apresentaram síndrome metabólica enquanto que nos pacientes com secreção autônoma de cortisol esse número subiu para 17%.

**Conclusão:**

A amostra de pacientes com tumores de adrenal e incidentaloma no HUGG apresentou uma prevalência de complicações metabólicas similares com a encontrada na literatura.

## INTRODUCTION

Adrenal Incidentaloma (AI) is defined as an asymptomatic mass, with a diameter greater than 1cm, discovered incidentally on abdominal examinations unrelated to the primary investigation of the adrenal. Although most adrenocortical tumors are benign and nonfunctional, approximately 5% to 30% are associated with hormone production. Advances in imaging methods have contributed to the increase in the detection of these adrenal masses^1^ ^,^ ^2^. However, lesions found during investigations in patients with established malignancy, high suspicion of malignant processes, or clinical evidence of adrenal disease should not be classified as incidentalomas^2^ ^,^ ^3^.

Determining the actual prevalence of AI is challenging, as it varies according to the characteristics of the population studied and the reasons for the imaging tests. Studies show that in autopsies, the prevalence varies from 0.4% to 2.1%, and can reach 7% to 8.7% in the elderly^3^ ^,^ ^4^. The frequency of AI is 3% in patients aged 50 years, increasing to 10% in the elderly. The prevalence of adrenal masses is 1.9% and 4.4% in patients without endocrine complaints and patients diagnosed with cancer, respectively^4^ ^-^ ^6^.

Most of the time, these tumors are benign and non-functioning, requiring no specific treatment; however, evaluation of the tumor’s potential malignancy may be warranted. Large neoplasms with associated clinical symptoms are referred for surgery^4^. However, the risk-benefit of surgery in patients with autonomic cortisol secretion still needs further studies, requiring an individualized approach.

Autonomic cortisol secretion, often associated with AI, can cause systemic conditions such as hypertension, diabetes mellitus, obesity, dyslipidemia, and osteoporosis/vertebral fractures. Although it does not present specific signs and symptoms, it differs from Cushing’s Syndrome^8^ ^-^ ^10^.

This study aims to analyze the cases of adrenal tumors followed up at the Gaffrée e Guinle University Hospital (HUGG), describing their clinical and epidemiological characteristics, as well as the clinical and metabolic profile of patients with adrenal tumors and adrenal incidentalomas treated at the HUGG.

## METHODS

This descriptive, cross-sectional study, approved by the HUGG Research Ethics Committee (opinion no. 198,899), covers patients treated at HUGG over the last three decades. Data collection involved the review of medical records in the endocrinology, general surgery, pathological anatomy, and Medical and Statistics Archive (SAME) services.

To ensure uniformity in data collection, a questionnaire was developed and applied between January and September 2020. The inclusion criteria were patients treated at HUGG since 1994, of any age and sex, with adrenal tumors. We selected medical records containing complete information for analysis of the clinical and metabolic profile of the tumors.

The tumors were classified into three groups based on size: <4cm, 4 to 6cm, and >6cm. The definition of functioning lesions was based on clinical and biochemical criteria already established in the literature^6^. The type of lesion was determined by the histopathological diagnosis in patients undergoing surgery, while in the others, hormonal evaluation and imaging characteristics were considered.

The metabolic profile was determined considering fasting glucose, glycated hemoglobin, lipid profile (total cholesterol, HDL, LDL, and triglycerides), blood pressure, BMI, diabetes mellitus, and obesity. Systemic arterial hypertension (SAH) was defined as a pressure ≥140x90mmHg on two occasions or a patient who was already being treated for it, while the criteria for prediabetes/diabetes mellitus (pre-DM/DM) followed the definition of the American Diabetes Association (ADA)^16^. Obesity was defined by the World Health Organization (WHO) as BMI >30.

For dyslipidemia, we considered LDL >160, TG >150, HDL <40, or treatment for dyslipidemia, according to the Brazilian guidelines. Metabolic syndrome was evaluated according to the WHO criteria^17^.

Statistical analysis was performed using Microsoft Office Excel 2016, and the results were discussed descriptively.

## RESULTS

**Graph 1:**
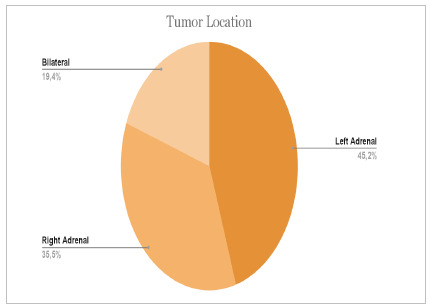
Tumor location

**Graph 2:**
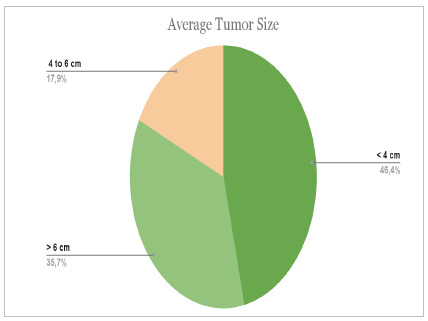
Average tumor size.

From a total of 31 medical records, we found 10 male patients (32%) and 21 female patients (68%). The mean age was 55 years, and the standard deviation was 16.2. Graphs 1 and 2 present the descriptive results of the location and size of the tumors found as a percentage.

**Graph 3:**
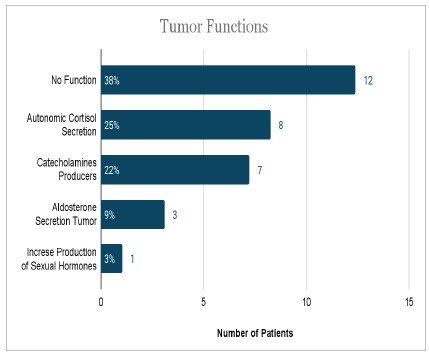
Numerical analysis and percentage of tumor functionality

**Graph 4:**
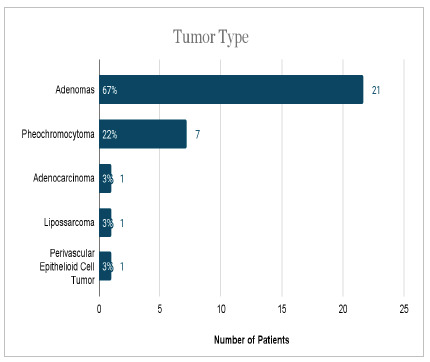
Numerical analysis and percentage of the tumor types.

In the sample, 17 patients (54%) had incidentalomas. In two cases, it was not possible to define whether they were incidentalomas according to the clinical and radiological history. Benign cases comprised 93.6% of the sample. Graphs 3 and 4 numerically present the results found when analyzing lesions’ types and functionality.

The mean size of lesions was 3.96 cm ± 2.09 cm for adenomas and 6.45 cm ± 3.4 cm for pheochromocytomas. Liposarcoma, pecoma, and adenocarcinoma represented single cases, with 15 cm, 10 cm, and 7 cm, respectively.

**Graph 5:**
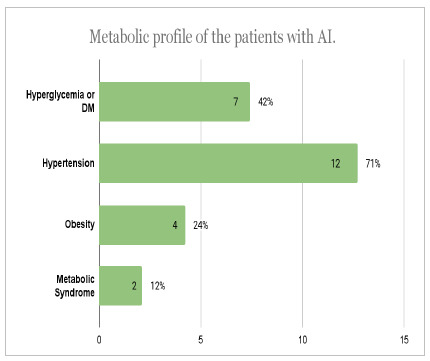
Metabolic profile of the patients with AI.

**Graph 6:**
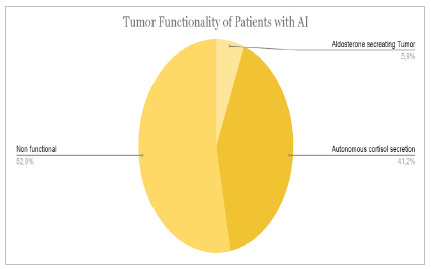
Tumor functionality of the patients with AI.

We also performed a specific analysis of patients with adrenal incidentaloma. A total of 17 patients were selected who met the AI criteria, i.e., an asymptomatic patient with an imaging test performed at random and without suspected malignancy. Graphs 5 and 6 show the prevalence of comorbidities and tumor characteristics in the sample of incidental patients. The data are presented in terms of absolute quantity and percentage of the sample cutoff.

The analysis of the profile of patients who present autonomy of cortisol secretion revealed six patients belonging to this group. Of these, four (67%) patients had SAH, four (67%) hyperglycemia or diabetes mellitus, two (33%) patients had dyslipidemia, and one (17%) patient had obesity and metabolic syndrome.

## DISCUSSION

Three epidemiological and physiological aspects can be analyzed to define and characterize tumors, namely, distribution according to sex, age, and location. Regarding the distribution by sex, the data of patients admitted to the HUGG show that 68% were female and 32% were male. This fact is in line with some studies, which reveal a predilection for the female sex^17^. A scenario of discrepancy occurs when we evaluate the location of the tumors, in which we observed a prevalence on the left side higher than the ones found in the literature (46% and 30-40%, respectively) and a lower prevalence in the right adrenal glands, of 35% and 50-60%^18^.

On the other hand, when evaluating age ranges, there is an agreement between our findings and the literature, showing in both a predilection for middle-aged patients. We found a mean of 55 years and a median of 59 years, which corroborates the description that the number of tumors increases between the 5th and 7th decade of life^3^ ^,^ ^18^.

Next, it is also worth highlighting the prevalence of adrenal tumors. Adenomas and adenocarcinomas maintain an expected frequency, as well as pheochromocytomas, when compared to a series of surgical cases8. In addition, to define the presence or absence of incidentaloma, we analyzed the clinical history of the patients and reached the values of 54% of individuals with suggestive history, 38% with clinical suspicion, and unverifiable history in 8%. This reality of increased incidentaloma diagnoses in recent decades is in line with the increasing use of better resolution imaging methods^11^.

The literature reinforces that the size of adrenal tumors varies according to etiology^2^. This premise was well demonstrated in this study, since the adenomas had a mean size of 3.96 cm. Pheochromocytomas, in turn, measured an average of 6.45 cm, which corroborates the result found in the literature, a mean size of 4.9 cm, with a variation of about 2.6 cm^19^.

Regarding the functionality of the studied adrenal tumors, 25% corresponded to cortisol-secreting tumors, 22% to catecholamines, 9% to aldosterone, and 3% to sex steroids, being a relevant part, expressing as clinically suggestive of CS, pheochromocytoma, and hyperaldosteronism. As for the group of patients (17) diagnosed with AI, a considerable portion had cortisol secretion of 41% - normal ranges from 12 to 29% -, a fact that can be justified by an autonomous cortisol secretion as subclinical Cushing’s Syndrome (SCS). 

When analyzing patients with cortisol secretion autonomy, 67% had hypertension, 67% hyperglycemia or DM, 33% dyslipidemia, and 17% obesity and metabolic syndrome. The values found for hypertension and DM are compatible with those found in the literature8. However, the prevalence values for obesity and dyslipidemia were, in part, different from those found in previous studies and, although high, they have been found on other occasions (68.8%)^18^. When evaluating dyslipidemias, we noticed a very variable prevalence in the references, with a value of 33% - the same as in the current study - evaluated in a previous study^19^. As for obesity, the prevalence found was lower than expected, although a similar result was observed on another occasion (19%)^20^.

We should highlight that in our study it was not possible to evaluate urinary albumin excretion and/or urinary albumin/creatinine ratio, and therefore the number of patients with metabolic syndrome may be underestimated according to the WHO criteria.

## CONCLUSION

Most the tumors in our series were incidentalomas, non-functioning adenomas. These data reinforce that the diagnosis of adrenal incidentalomas is important because these lesions can be associated with metabolic alterations such as hypertension, obesity, diabetes mellitus, and dyslipidemias, with metabolic complications being more pronounced in patients with autonomy of cortisol secretion.

The diagnostic work-up of adrenal incidentaloma includes laboratory tests to differentiate functioning from non-functioning lesions. Autonomic cortisol secretion is a hormonal disorder detected almost exclusively in the context of adrenal incidentaloma and may be related to several systemic conditions.
